# Factors Associated with Survival to Discharge in Cats with Spontaneous Hemoperitoneum

**DOI:** 10.3390/ani16162566

**Published:** 2026-08-17

**Authors:** Douglas Evans, Alexandra Guillén, Stefano Cortellini, Matteo Rossanese

**Affiliations:** Department of Clinical Science and Services, The Royal Veterinary College, Hatfield AL9 7TA, UK

**Keywords:** Hemoabdomen, splenectomy, neoplasia, feline, liver, hemangiosarcoma

## Abstract

This study aims to describe the clinical presentation, treatment and outcomes of cats diagnosed with spontaneous hemoperitoneum and to identify factors associated with survival to discharge. Medical records from 2005 to 2024 were searched and data was reviewed retrospectively. Clinical, diagnostic and treatment variables were evaluated for their association with survival to discharge. Seventy-three cats were included. The most common presenting clinical signs at diagnosis were lethargy, hyporexia and collapse. Hemoperitoneum was most often originating from the liver (41 cats), the spleen (19 cats) and pancreas (three cats), with forty-two cats requiring transfusion of blood products. Twenty-two cats (30%) underwent surgery while 21 cats (29%) were managed conservatively, and 30 (41%) cats were euthanized at presentation or diagnosis. Neoplasia was the most common diagnosis (50 cats, 68.5%) with hemangiosarcoma being most common. The most common non-neoplastic conditions included amyloidosis (eight cats) and hepatic vacuolar hepatopathy (three cats). Overall survival to discharge was 38%. Surgery was the only variable associated with survival to discharge (*p* = 0.031). Cats receiving transfusions were more likely to survive (*p* = 0.013). Survival did not differ between cats with and without neoplasia (*p* = 0.366) or by bleeding origin (liver vs. spleen; *p* = 0.483). Survival to discharge for cats with spontaneous hemoperitoneum remains guarded. Hepatic and splenic neoplasia were the most common diagnoses, with hemangiosarcoma representing the most common primary neoplasia. Cats undergoing surgery, regardless of final diagnosis, and those receiving blood products had the highest likelihood of survival to discharge.

## Simple Summary

Spontaneous internal bleeding into the abdomen is an uncommon but serious condition in cats and can be life-threatening. Information about the causes, treatment options, and outcomes of affected cats is limited. This study reviewed the medical records of 73 cats diagnosed with spontaneous abdominal bleeding over a 19-year period to better understand how these cats present, what conditions cause the bleeding, how they are treated, and which factors influence survival to discharge. The most common signs seen by owners were reduced appetite, lethargy, and collapse. Tumors were the most frequent cause of bleeding, particularly those affecting the liver and spleen. Other causes included conditions that altered the structure or function of the liver. Almost one-third of the cats underwent surgery, while others were managed medically or were euthanized because of the severity of their condition or a perceived poor prognosis. Cats undergoing surgery had a higher rate of survival to discharge, although the retrospective nature of the study means that surgery cannot be assumed to have caused this improvement, as cats selected for surgery may have been considered better surgical candidates. Blood transfusions were more common among cats undergoing surgery but were not independently associated with survival. No significant association was identified between survival and either the neoplastic or non-neoplastic nature of the underlying disease or the anatomical source of bleeding. Bleeding site was also not significantly associated with the decision to pursue surgery. These findings provide valuable information for veterinarians and cat owners when making treatment decisions and discussing prognosis for cats with spontaneous abdominal bleeding.

## 1. Introduction

Hemoperitoneum is defined as an accumulation of blood into the peritoneal space, and it is categorized according to the etiology as traumatic or spontaneous [1,2,3,4,5]. Spontaneous or non-traumatic hemoperitoneum has been associated with many pathological processes including benign or malignant intra-abdominal neoplasia, coagulopathies, gastrointestinal ulcer perforation, vena cava syndrome secondary to dirofilariasis, hepatic amyloidosis, anaphylaxis, portal vein thrombosis, pancreatitis, leptospirosis-induced hepatitis and intra-abdominal organ torsion [1,2,3,4,5,6,7,8,9]. The most common cause of spontaneous hemoperitoneum in dogs is malignant neoplasia (68–87%), with the source of bleeding most commonly arising from the spleen (62.5–90%), most often due to hemangiosarcoma (HSA) (47–88%) [3,5,6,7,9,10,11,12]. Other reported sources of hemorrhage include the liver, kidneys, omentum, urinary bladder, gastrointestinal tract, pancreas, prostate gland, mesentery, abdominal lymph nodes, adrenal glands and peritoneum [1,4,5,7,11,13]. Spontaneous hemoperitoneum is a rare life-threatening condition in cats. In one study hemorrhage was hepatic in origin in 75% of cats, with 42% of these being neoplastic in origin [1]. In a larger study, a non-neoplastic condition was the cause of hemoperitoneum in 54% of cats, including coagulopathies and hepatic necrosis, with the liver being the most common origin of the hemorrhage (40%) for both neoplastic and non-neoplastic causes [4]. Despite the even distribution between neoplastic and non-neoplastic diseases, the prognosis has been reported to be poor, with only 12% of cats surviving hospital discharge [1,4]. Of the 65 cats in one study, 50 were euthanized and 15 died by the end of the study period [4]. In more recent studies, the outcome seems to have improved with rates of survival to discharge ranging from 48% (16/33 cats) to 75% (6/8 cats), although higher survival rates in those studies could have been influenced by the inclusion of cats with traumatic and iatrogenic hemoperitoneum [14,15]. The prognosis for spontaneous hemoperitoneum in cats appears poor; however, relatively scarce information exists in the veterinary literature and further investigation is warranted. The objectives of this study were (1) to describe the clinical presentation, treatment and outcomes of cats diagnosed with spontaneous hemoperitoneum, (2) to record the incidence of malignancy, and (3) to identify prognostic factors for survival to discharge.

## 2. Materials and Methods

This retrospective study used anonymized clinical data and was approved by the social science research ethical review board of the Royal Veterinary College, University of London (approval no.: URN SR2022-0070). Clinical records from The Queen Mother Hospital for Animals, Royal Veterinary College were searched from January 2005 to February 2024 to identify cats that had been diagnosed with spontaneous hemoperitoneum.

Cats diagnosed with spontaneous hemoperitoneum during the study period that had comprehensive clinical records were included in the study. Hemoperitoneum was defined as an accumulation of blood within the peritoneal cavity as determined by a fluid analysis performed following abdominocentesis or on the basis of either grossly visible blood within the peritoneal cavity at the time of surgery, or at necropsy [3,5,6,16]. Exclusion criteria included incomplete clinical records, cats with hemoperitoneum attributed to iatrogenic causes (e.g., surgery, abdominocentesis, biopsies) or with a recent history of traumatic injury (<1 month prior to presentation to the authors’ institution).

### 2.1. Data Extraction

Two investigators (DE, MR) independently searched the database of the Royal Veterinary College, using the search engine available in the practice management systems (CRIS™), searching for the keywords “hemoabdomen”, “hemoperitoneum” with “feline”, and “cat”. The search was conducted between March 2024 and May 2024.

Information retrieved from the records included signalment; presenting clinical signs; physical examination findings; preoperative blood test results; preoperative diagnostic findings; surgical treatment (when applicable); peritoneal fluid analysis; cytological and histopathological findings; time from presentation to discharge; survival to hospital discharge; and postoperative treatments. Anemia was considered as a pack cell volume (PCV) of less than 25% [17]; thrombocytopenia was defined as a platelet count of less than 200 × 10^9^/L confirmed with blood smear evaluation. Shock index (SI) was calculated as the ratio between the heart rate and Doppler systolic blood pressure [18]. The inciting cause of hemoperitoneum and the anatomical source of hemorrhage were determined on the basis of clinical, diagnostic and histopathological findings and predefined as splenic, hepatic, other location (i.e., retroperitoneum, kidney, adrenal, other organs), multiple organs, or undetermined [7]. Post-discharge follow-up was obtained by review of electronic patient records from the referral hospital and referring veterinary practice. When appropriate, documented tumor progression and follow-up information was obtained by review of electronic patient records from the referral hospital and contacting referring veterinarians and/or owners via telephone. When a tumor was diagnosed, tumor progression was defined as evidence of recurrence of a mass documented on imaging investigations or development of nodal or distant metastasis as confirmed to be associated with the initial diagnosis by cytology or histopathology. The cause of death was classified as either related or unrelated to the hemoperitoneum-causing disease. Disease-related deaths were further classified as associated either with surgical complications or with the disease itself.

### 2.2. Statistical Analysis

Analyses were performed using Microsoft Excel (version 14.00; Microsoft Corp, Redmond, WA, USA) and SPSS 29.0 (IBM SPSS statistics, version 29.0; IBM Corp, Armonk, NY, USA). Descriptive statistics were computed for all variables. Categorical variables were described as frequency and percentages. Continuous variables were tested for normality using the Shapiro–Wilk test. If normally distributed, data were summarized as mean and standard deviation (+/−SD). If non-normally distributed, data were summarized using median and interquartile range (IQR).

Outcomes of interest for each cat included diagnosis, survival to discharge, and survival time. All times were calculated from the date of surgery to the event (discharge or death) or censorship. Cats were censored from analysis if they were alive at the time of analysis, died for reasons unrelated to the source of hemoperitoneum, or were lost to follow-up. The cause of death was classified as either related or unrelated to the hemoperitoneum-causing disease.

All cats meeting the inclusion criteria were included in the descriptive analysis. However, cats euthanized immediately following diagnostic investigation, before either surgical or conservative treatment was attempted, were excluded from comparative statistical analysis evaluating treatment outcome. Comparative analysis therefore included only cats managed surgically or conservatively.

Differences between cats that survived to discharge and those that did not, and between cats that underwent surgery and those that did not, were assessed using Fisher’s exact test for categorical variables (gender, collapsing episodes, respiratory signs, comorbidities, tachycardia, hypoalbuminemia, received transfusion, bleeding origin [spleen vs. liver], diagnosis [neoplasia vs. non-neoplasia], and surgery status). Continuous variables (age, body weight, body condition score, shock index, duration of clinical signs, and packed cell volume) were compared using the Mann–Whitney U test. A *p* value < 0.05 was considered statistically significant.

## 3. Results

### 3.1. Demographics and Clinical Presentation

**Figure 1:**
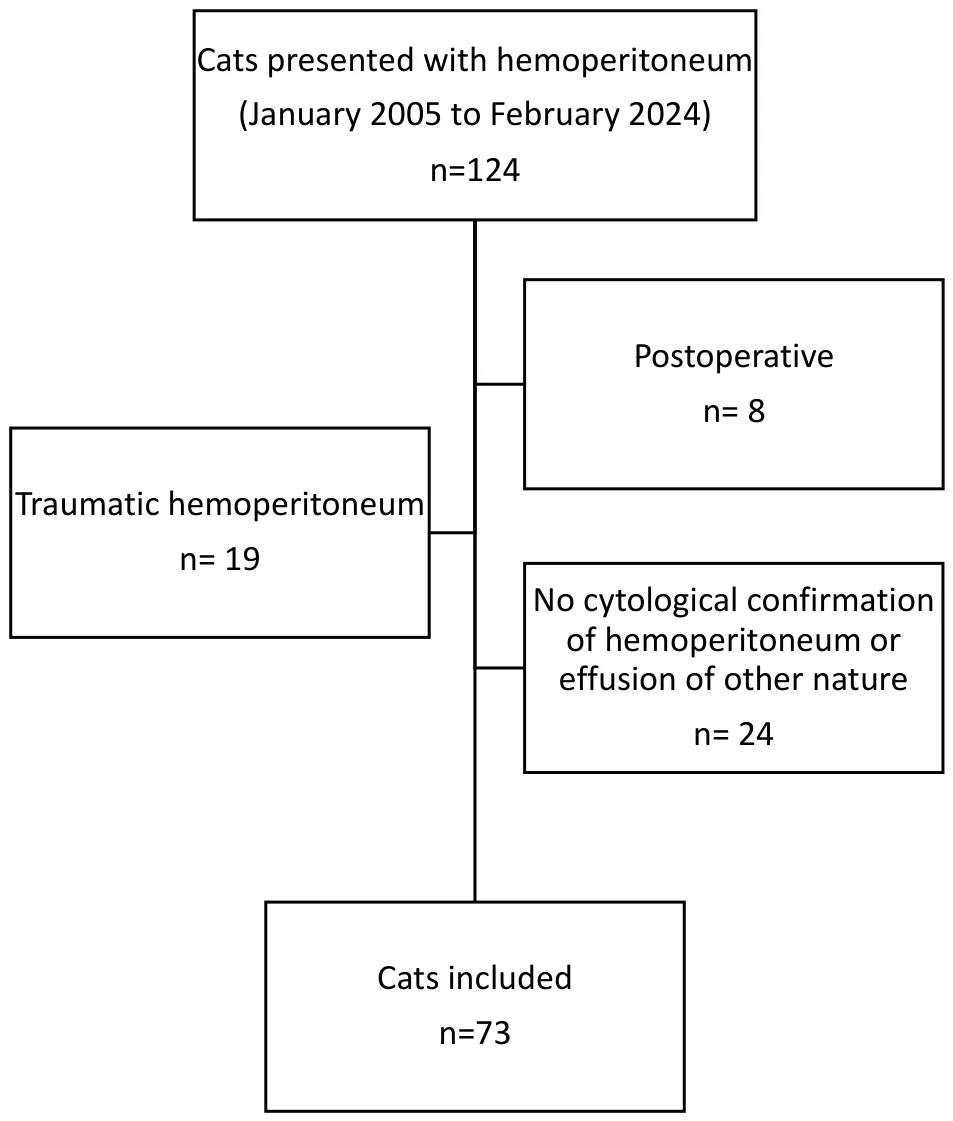
Case inclusion algorithm for cats with spontaneous hemoperitoneum.

Seventy-three cats met the inclusion criteria (Figure 1).

The population included 40 male cats (39 neutered; one entire) and 33 female cats (31 neutered; two entire). The most represented breed was domestic shorthair (45) followed by Siamese (eight), domestic longhair (five), Bengal (five), British shorthair (three), Siberian (one), oriental (one), Chantilly Tiffany (one), Turkish Van (one), Birman (one), Abyssinian (one), and British longhair (one). At the time of diagnosis median body weight was 4.6 kg (IQR, 3.6–5.4), median age was 9 years (IQR, 9–12), and median body condition score was 4 (IQR, 3–5).

Main clinical signs at presentation and physical examination findings are summarized in Table 1. In most cats, a combination of clinical signs was reported. Median duration of clinical signs at the time of first presentation was 3 days (IQR, 1–7) and median shock index was 1.86 (range 1–3.33, data available for 48 cats). Twenty-one cats (29%) had co-morbidities at the time of diagnosis, the most common being: hyperthyroidism (four), chronic kidney disease (four), gingivostomatitis (four), inflammatory bowel disease (three) and hypertrophic cardiomyopathy (two). Apart from one cat with hematochezia, no other cats showed any evidence of spontaneous external bleeding.

### 3.2. Diagnostic Investigations

Complete blood count was available in 50 cats and serum biochemistry in 55 cats; the most common abnormalities are included in Table 2. Serum biochemistry was within the reference limits in 20 cats (36%). Feline leukemia virus and feline immuno-deficiency virus in tests were negative in the 19 cats tested, except one cat which tested positive for feline immuno-deficiency virus. As part of the initial stabilization, 41 cats received blood products, and 12 of them received more than one blood product, including xenotransfusion (canine packed red blood cells) (16), feline fresh whole blood (nine), Oxyglobin™ (seven), autotransfusion (10), feline fresh frozen plasma (nine), and feline pRBC (five). All cats underwent abdominocentesis, and hemoperitoneum was diagnosed in all cats with a median abdominal fluid PCV of 20% (IQR, 15–26). Abdominal imaging reports were available for review in 71 cats, including abdominal ultrasound in 57 cats, thoracic and abdominal CT in 19 cats, and thoracic and abdominal radiographs in 18 cats. Echocardiography was performed in nine cats. A combination of these imaging modalities was used in 23 cats. All diagnostic imaging was performed by, or under the direct supervision of, a European or American College of Veterinary Diagnostic imaging (ECVDI-ACVDI) board-certified radiologist. The most common diagnostic findings suspected to be related to the hemoperitoneum were a hepatic mass (20), nodular/multifocal hepatopathy (19), splenic mass (18), hepatomegaly (four), mid-abdominal mass (four), splenic nodules (two), renal mass (two) and retroperitoneal mass (one). Thirty-two lesions were sampled via fine needle aspiration. Cytological samples revealed hepatic amyloidosis (nine), hepatic neutrophilic inflammation (five), vacuolar hepatopathy (three), cholestasis (two), splenic HSA (two), hepatocellular carcinoma (two), reactive splenopathy and extramedullary hematopoiesis (one), pancreatitis (one), suspected pancreatic carcinoma (one), splenic hematoma (one), severe suppurative hepatitis (one), suspected splenic sarcoma (one), splenic mast cell tumor (one), pancreatic malignant neoplasia (one), and splenic mesenchymal neoplasia (one). Three samples were not diagnostic. Based on the diagnostic investigations, the suspected source of the hemoperitoneum was the liver (41), spleen (19), pancreas (three), both the spleen and liver (two), kidney (two), abdominal mass (two), caecum (one), and retroperitoneum (one). In two cats, the cause could not be determined.

### 3.3. Diagnosis, Surgical Management, and Outcomes in Cats Undergoing Surgery

**Figure 2:**
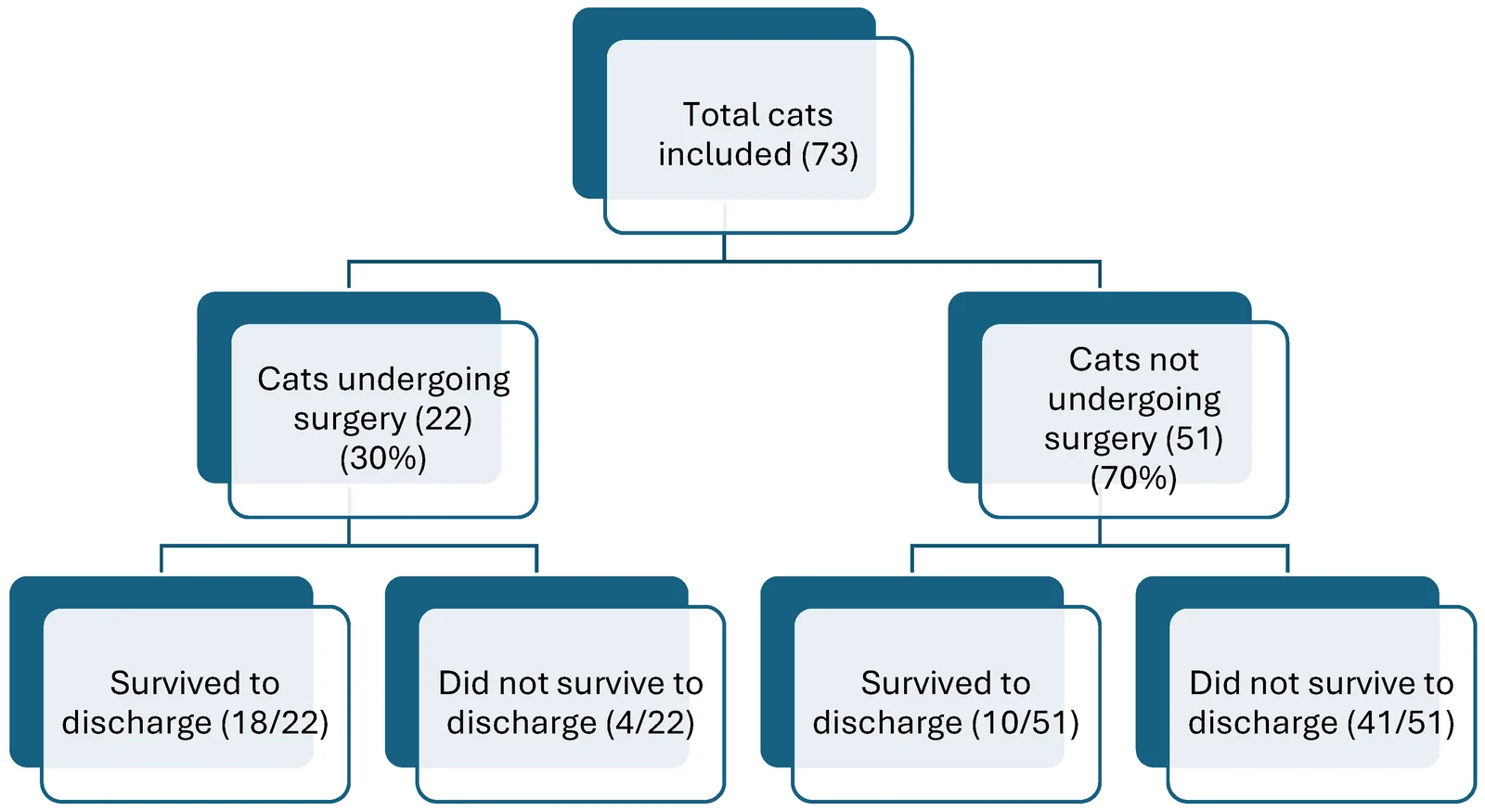
Survival to discharge in cats with spontaneous hemoperitoneum according to management strategy.

Twenty-two (30%) cats underwent exploratory celiotomy with splenectomy being the most common procedure performed (10) followed by liver lobectomy (eight), excision of multiple hepatic lesions (one), ileocecocolic junction enterectomy (one), ureteronephrectomy (one), and ureteronephrectomy and adrenalectomy (one). Ten concomitant procedures were performed in 10 cats including liver biopsies (five), regional lymphadenectomy (two), partial pancreatectomy (one), gastrostomy tube placement (one), and nasogastric feeding tube placement (one). Nineteen cats received a blood transfusion. All surgeries were performed by, or under the direct supervision of, a European or American College of Veterinary Surgeons (ECVS or ACVS) board-certified surgeon. Intraoperative complications were observed in two cats: one cat suffered a period of hypotension managed with a combination of crystalloids and colloids; in one cat, undergoing ureteronephrectomy and adrenalectomy, the caudal vena cava was damaged, causing bleeding and hypotension, and the contralateral ureter was accidentally severed and needed a neoureterocystostomy. This cat received Oxygobin^TM^ and a Jackson Pratt active suction drain was also placed. During the postoperative period, one cat undergoing liver lobectomy deteriorated and was euthanized. Six cats received further blood products due to bleeding, hypotension and persistent anemia during hospitalization including Oxyglobin™ (one), xenotransfusion (canine packed red blood cells) (three), and feline fresh whole blood (two). Median hospitalization period was 5 days (IQR, 4–7). Histopathological evaluation was available in all cats revealing 17 neoplastic and five non-neoplastic lesions (Table 3). Neoplastic diagnoses included: hemangiosarcoma (10; 7 splenic, two hepatic, one cecal), hepatic adenoma (two), HCC (one), splenic mast cell tumor (one), poorly differentiated splenic sarcoma (one), mesenchymal renal neoplasia (one), and biliary carcinoma (one). Benign lesions included: hematoma/vacuolar hepatopathy (three), splenic hematoma/nodular hyperplasia (one), and bilateral polycystic kidney disease (one). Metastasis at the time of surgery was identified in four cats: three cats with splenic HSA had metastasis to the liver (two) and regional lymph nodes (two), and one cat with poorly differentiated sarcoma had metastasis to the liver. Eighteen (82%) cats survived to discharge (Figure 2).

Of the four cats that died during hospitalization, two had a neoplastic condition (splenic HSA with metastasis to the liver and regional lymph nodes and a splenic poorly differentiated sarcoma with metastasis to the liver) and two had a non-neoplastic condition (hepatic hematoma with extramedullary hematopoiesis and a splenic hematoma with nodular lymphoid hyperplasia). All four cats were euthanized due to continued clinical deterioration during hospitalization. Median time of follow-up for the 18 cats surviving to discharge was 387 days (range, 35–1710). Six cats received chemotherapy postoperatively, with all of them being diagnosed with visceral HSA (four spleen, one liver, one caecum). First-line treatments included: doxorubicin and cyclophosphamide (two), metronomic chlorambucil, doxorubicin, masitinib, and toceranib (one each). During the follow-up period four cats underwent complete restaging between 60 and 754 days after surgery; local recurrence was reported in one cat with hepatic adenoma 790 days after surgery that underwent a second liver lobectomy. Distant metastases were reported in one cat with splenic HSA with metastasis to the liver diagnosed 927 days from surgery. Four cats with hepatocellular adenoma (two), hepatic hematoma and cholangiocellular carcinoma (one each) were still alive at the time of the study writing. Three cats were lost to follow-up between 30 and 384 days (splenic MCT, cecal HSA, polycystic kidney disease). Eleven cats died or were euthanized at between 35 and 1546 days following discharge; this was related to the primary cause of the initial hemoperitoneum in seven cats.

### 3.4. Diagnosis and Outcomes in Cats Not Undergoing Surgery

Fifty-one cats did not undergo surgery; median hospitalization period was 2 days (IQR, 1–3). Of the 51 cats, twenty-three cats received a presumptive diagnosis of neoplasia based on diagnostic imaging findings only, and further investigations were either not pursued or declined. Seventeen cats underwent cytological assessment: in nine cats, a non-neoplastic condition was identified, whereas neoplasia was confirmed in eight. In an additional eight cats, the diagnosis was obtained post-mortem; five had non-neoplastic condition and three had neoplasia. In two cats the origin of the hemoperitoneum was unknown. In 10 cats, metastatic disease was suspected at the time of investigations in the liver (six), regional lymph nodes (three), spleen (two), and lungs (one).

Thirty-one cats (42%) were euthanized at presentation or following diagnostic investigation based on veterinary recommendation or owner request due to the severity of clinical signs, diagnostic findings, perceived poor expected outcome (i.e., hepatic and splenic mass) or limited treatment options.

Twenty-one (29%) cats underwent diagnostic investigation and initial medical stabilization, and the hemoperitoneum was treated conservatively. Management decisions were made on an individual-case basis, considering the underlying condition, clinical status, and the preferences of the attending clinician. Median hospitalization period was 3 days (IQR, 4–7).

Of these cats, eleven did not survive to hospital discharge: two experienced cardiopulmonary arrest; cardiopulmonary resuscitation was successful in one, but the cat was subsequently euthanized. The remaining nine cats were euthanized due to clinical deterioration. Ten (20%) cats survived to discharge (Figure 2) and follow-up ranging from 39 to 1101 days. Of these, six had a non-neoplastic condition and four had a neoplastic condition (three presumed, one confirmed). Four cats were lost to follow-up, one cat was still alive at the end of the study period, and five cats were euthanized for causes suspected to be associated with the hemoperitoneum. The specific diagnoses are summarized in Table 4.

### 3.5. Factors Associated with Survival to Discharge

When comparing cats that did and did not undergo surgery, those undergoing surgery were significantly more likely to present in a collapsed state (*p* = 0.023) and more likely to receive a blood transfusion (*p* = 0.013) (Table 5). Overall, undergoing surgery was associated with a significantly higher probability of survival to discharge compared with not undergoing surgery (*p* = 0.031). There was no difference in survival to discharge between cats diagnosed with neoplasia and those without neoplasia (*p* = 0.366), nor was survival influenced by the origin of bleeding (*p* = 0.483) (Table 5).

## 4. Discussion

The results of this study indicate that while survival to discharge remains guarded for cats with hemoperitoneum, the rate observed in this study (38%) was higher than previously reported [1,4,14]. Hepatic (33%) and splenic (27%) neoplasia were the most common diagnoses in cats presenting with hemoperitoneum in this cohort, with HSA representing the most common primary neoplasia. In this study, the liver was the primary source of bleeding, followed by the spleen. This distribution is consistent with previous reports in cats [1,4] but contrasts with the situation in dogs, where the spleen is the most commonly affected organ in cases of spontaneous hemoperitoneum [3,5,6,7,9,10,11,12].

Among cats with histopathology available, neoplasia accounted for 68% of cases. Hepatic (33%) and splenic (27%) neoplasia were the most common diagnoses in cats presenting with spontaneous hemoperitoneum in this cohort, with HSA representing the most common primary neoplasia (23% of all cases; 52% of neoplastic cases). This represents a higher proportion of neoplasia than previously reported by Culp et al. (2010) [4] and Mandell and Drobatz (1995) [1], who found a similarly even distribution between neoplastic (44–46%) and non-neoplastic (54–56%) causes. In the current study, the overall survival to discharge was 38%, which is better than the 12% previously reported [1,4]. This improvement is likely multifactorial. Advances in veterinary medicine, including early diagnosis, increased blood product availability for cats and management of hemoperitoneum, have improved outcomes. Likely, changes in clients and veterinary professionals’ perception of treatment of neoplastic diseases in cats might have also affected consideration for further treatment in cats with hemoperitoneum and potential underlying cancer. Differences in study populations, referral patterns, case selection, underlying etiologies and euthanasia practices may also account for some or all of the variation between studies.

However, it is worth to mention that cats in this study were recruited over a broad timespan (2005–2023). Our feline blood bank was established in 2017, and case management has evolved significantly since then. Interestingly, our analysis did not show any difference in outcome based on year of presentation. Among cats that did not survive to discharge, 43 of 45 cats were euthanized during hospitalization due to clinical deterioration and/or perceived poor prognosis. However, definitive histopathological diagnosis was only obtained in 30 cats, complicating assessment of underlying disease. The retrospective nature of this study presents challenges in evaluating the factors influencing euthanasia decisions. It is plausible that imaging findings suggestive of neoplasia, combined with the historically poor prognosis for hepatic and splenic tumors, contributed to these decisions. Cats undergoing surgery had higher survival to discharge (82%) compared to those that did not (18%). This finding is consistent with Bunnel et al. (2023) who reported a survival to discharge of 78% (7/9 cats) for all cats undergoing surgery for various causes of hemoperitoneum [14]. This is not surprising as the successful management of hemoperitoneum includes correction of hypovolemia, patient stabilization, rapid identification and control of the bleeding source, and adequate post-operative management [16,19]. In this study, surgery was the only factor significantly associated with survival to discharge. However, this association should not be interpreted as evidence of a causal effect, as cats undergoing surgery were a selected population and were likely considered more suitable surgical candidates. The findings may therefore reflect both the potential benefit of controlling the source of hemorrhage and differences in disease severity, surgical feasibility, clinician recommendations and owner decision-making between cats that did and did not undergo surgery.

Unexpectedly, cats undergoing surgery were more frequently collapsed on presentation, suggesting that clinical status did not necessarily influence clinician and owner decisions against surgical intervention or rendered surgery contraindicated due to perceived poor prognosis. Given that surgery was not performed in the majority of cats that were euthanized, this highlights the approach to feline hemoperitoneum clients that veterinarians might have or had had in the past towards potential neoplastic diseases, suggesting that the major mortality factor was the decision against pursuing any additional treatment, including surgery. In addition, undergoing surgery could have reflected the systemic nature of the underlying condition; some conditions leading to hemoperitoneum in cats, such as amyloidosis or degenerative liver disease, might not be amenable to surgical treatment and in these cases, euthanasia could have potentially been offered early in time. In the present study, the anatomical source of bleeding was not significantly associated with either the decision to pursue surgery or survival to discharge. Although splenectomy may be perceived as more straightforward than liver lobectomy, particularly when hepatic lesions are located in surgically challenging regions, this distinction did not appear to influence case management or short-term outcome in this cohort. These findings suggest that bleeding site alone should not be used to determine surgical candidacy or prognosis; instead, decisions should be based on the individual patient’s clinical status, underlying disease and feasibility of surgical intervention.

Receiving a transfusion was a factor more frequent in the surgical group, which may reflect the intent to proceed with further diagnostics and surgical intervention, as well as efforts to stabilize the cat’s cardiovascular status in view of ongoing management. In the study by Bunnell et al. (2023) [14], transfusion administration was significantly associated with a better outcome, with cats that received a transfusion demonstrating higher survival to discharge rates (71% vs. 31%). Similarly, Cole and Humm (2018) reported encouraging outcomes in a small cohort of cats with hemoperitoneum receiving autologous transfusions, with three of four cats with spontaneous cases surviving to discharge [15]. A separate study in dogs has indicated that administering blood products early during the management of hemorrhagic shock can improve survival, likely through enhanced tissue oxygen delivery and perfusion [20], which may help explain the improved outcomes seen in transfused cats. Moreover, it is plausible that cats receiving transfusions were also more likely to undergo additional recommended treatments, contributing further to the increased survival observed in this group.

It is difficult to directly compare outcomes across studies, given the heterogenous etiologies in feline hemoperitoneum. For example, in the study by Cole and Humm (2018) evaluating the use of autologous blood transfusion in cats with hemoperitoneum, of four cats with spontaneous hemoperitoneum, three survived to discharge, though one was subsequently lost to follow-up, and two were euthanized within 6 weeks due to progressive disease [15]. In contrast, Bunnell et al. (2023), reported that of the 17 cats with an underlying neoplastic disease, out of the 33 described presenting with spontaneous hemoperitoneum, 15 did not survive to discharge [14].

A review of clinical records from our cohort indicated that the study by Culp et al. (2010) was frequently cited in decision-making discussions regarding euthanasia, with some cats euthanized on the perception of universally poor prognosis, irrespective of specific etiology [4]. However, significant advancements in veterinary critical care, surgical techniques, and the availability of feline blood products have improved outcomes for conditions previously considered fatal. Therefore, clinicians should carefully evaluate each case individually rather than relying solely on historic data, as prognosis may vary considerably depending on the underlying cause and the patient’s response to modern therapeutic interventions.

While this study assessed only short-term survival, external data may help contextualize longer-term outcomes. Brandstetter et al. (2023) [21] and Hylton et al. (2026) [22] reported that, when indicated, liver lobectomy is an effective treatment option for cats with primary non-hematopoietic malignant liver tumors, achieving prolonged survival times (MST of 375–673 days) for hepatocellular carcinoma and biliary carcinoma. However, those studies did not specifically address cats with hemoperitoneum, and thus the prognosis for such cases remains uncertain [21,22].

A recent study evaluating splenectomy in cats reported an overall MST of 136 days for cats diagnosed with splenic neoplasia (348 days for mast cell tumor and 94 days for HSA), while cats with non-neoplastic splenic processes had an MST of 715 days [23]. In that cohort, cats with HSA were more likely to present with hemoperitoneum, although this did not impact overall outcome. The prognosis for cats with splenic HSA remains poor, with MSTs ranging from 77 to 197 days [4,23,24,25], and all reported cases of cats ultimately resulted in death or euthanasia due to disease progression. It is important to provide owners with this information to enable informed decision-making regarding the continuation of intensive care.

Conservative management may be a reasonable option for cats, particularly when clinical signs are mild and patients remain hemodynamically stable. Careful monitoring and supportive care can result in favorable outcomes while avoiding the risks associated with surgical intervention, particularly in non-neoplastic cases. However, this approach is often independent of the definitive diagnosis, which remains challenging to establish and frequently requires some form of invasive investigation. Despite these challenges, conservative management of non-neoplastic conditions can be beneficial as demonstrated by the 20% survival of the cats medically managed, and further studies are needed to improve diagnostic accuracy and guide optimal treatment strategies in these patients. This study is limited by its retrospective design and reliance on medical records, which may have been incomplete or inconsistent and not be able to elucidate the reasoning behind patients’ management. Treatment allocation was not randomized, and the association between surgery and improved survival is therefore subject to substantial selection bias and confounding by indication. Cats selected for surgery were likely considered better surgical candidates based on their clinical condition, suspected underlying disease, anatomical feasibility, clinician assessment and owner preference. In contrast, cats with more severe disease, conditions not amenable to surgical treatment or a perceived poor prognosis may have been less likely to undergo surgery and more likely to be euthanized. Consequently, the observed association should not be interpreted as evidence that surgery independently caused improved survival.

Euthanasia without full diagnostic investigation or attempted treatment likely introduced bias, particularly leading to underestimation of survival. Histopathological confirmation was available in only 41% of cases, limiting conclusions about underlying etiologies.

The single-center referral population, combined with a long study period spanning advances in critical care, anesthesia, and transfusion medicine, may also limit generalizability. Finally, survival was assessed only to hospital discharge, precluding evaluation of long-term prognosis.

## 5. Conclusions

Spontaneous hemoperitoneum in cats carries a guarded prognosis, but survival to discharge is higher than previously reported. Surgical intervention was the only independent positive prognostic indicator, while transfusion may provide supportive benefit. Aggressive management should not be withheld solely due to suspected neoplasia or bleeding site, as neither was independently associated with outcome. These findings may help clinicians guide treatment decisions and provide owners with more accurate information about the expected outcome.

## Figures and Tables

**Table 1:** Summary of clinical signs and physical examination findings in cats presented with spontaneous hemoperitoneum.

Clinical Signs	Nº of Cats (%)
Lethargy	52/73 (71%)
Hyporexia/anorexia	30/73 (41%)
Collapsing episodes	20/73 (27%)
Vomiting	12/73 (16%)
Tachypnea/Dyspnea	11/73 (15%)
Weight loss	9/73 (12%)
Weakness	8/73 (11%)
Depression/abnormal behavior	6/73 (8%)
Abdominal distension	5/73 (7%)
Polyuria/polydipsia	4/73 (5.5%)
Ataxia	3/73 (4%)
Diarrhea	2/73 (2.8%)
Adipsia	1/73 (1.4%)
Ptyalism	1/73 (1.4%)
**Physical examination findings**	
Pale mucous membrane	52/73 (71%)
Distended abdomen	35/73 (48%)
Heart murmur	28/73 (38%)
Peripheral pulse deficits	27/73 (37%)
Abnormal behavior	22/73 (30%)
Palpable abdominal mass	13/73 (18%)
Gallop rhythm	12/73 (16%)
Dehydration	7/73 (9%)
Icterus	5/73 (7%)
**Vital parameters**	
Tachycardia (>220 bpm)	14/70 (20%)
Bradycardia (<140 bpm)	7/70 (10%)
Tachypnea/dyspnea (>40 bpm)	35/69 (51%)
Hypothermia (<37.5 degrees)	29/65 (45%)
Hyperthermia (>39.2 degrees)	2/65 (3%)
Hypotension (<90 mmHg)	15/48 (31%)

**Table 2:** Main clinico-pathological abnormalities in cats with spontaneous hemoperitoneum.

Parameters	Nº Cats	Median Value (Range)	Normal Reference Range
PCV	73/73 69 (95%); 41 regenerative	16% (7–32)	25–45%
Anemia			
Total proteins	53/73 17 (32%)	61 g/L (35–95)	60–80 g/L
Hypoproteinemia			
Albumin	53/73 15 (28%)	26 g/L (16–36)	25–45 g/L
Hypoalbuminemia			
ALT	53/73 39 (74%)	291 U/L (28–5394)	5–60 U/L
Elevated			
ALP	51/73 6 (12%)	21 U/L (8–916)	0–60 U/L
Elevated			
Total bilirubin	42/73 26 (62%)	6.4 umol/L (0–230)	0.1–5.1 umol/L
Hyperbilirubinemia			
Urea	50/73 21 (44%)	12.2 mmol/L (4.7–57.8)	2.5–9.9 mmol/L
Elevated			
Creatinine	50/73 11 (22%)	107 umol/L (24–525)	20–177 umol/L
Increased			
Platelets	55/73 14 (25%)	* (22–986)	200–800 × 10^9^/L
Thrombocytopenia			
Lactate	35/73 31 (89%)	4.2 mmol/L (0.8–17)	0.6–2.5 mmol/L
Hyperlactatemia			
Neutrophils	43/73 28 (65%)	14.3 × 10^9^/L (3.9–39)	2.5–12.5 × 10^9^/L
Neutrophilia			
WBC	43/73 17 (40%)	16.9 × 10^9^/L (5.5–45.9)	5.5–19.50 × 10^9^/L
Leukocytosis			
Prothrombin time	36/50 6 (17%)		7–11 s
Elevated			
Activated thromboplastin time	36/50 16 (44%)		13–20 s
Elevated			

* Median platelets count not reported due to unreliable automated values; adequacy was confirmed by blood smear evaluation.

**Table 3:** Origin, diagnosis and outcomes of 22 cats with spontaneous hemoperitoneum undergoing surgical management.

Bleeding Origin	Diagnosis	Method of Diagnosis	Not Surviving to Discharge
Spleen (10)	Hemangiosarcoma (7)	Histopathology (7)	1 cat
	Mast cell tumor (1)	Histopathology (1)	
	Poorly differentiated sarcoma (1)	Histopathology (1)	1 cat
	Hematoma/nodular hyperplasia (1)	Histopathology (1)	1 cat
Liver (9)	Hemangiosarcoma (2)	Histopathology (2)	
	Adenoma (2)	Histopathology (2)	
	Hepatocellular carcinoma (1)	Histopathology (1)	
	Biliary carcinoma (1)	Histopathology (1)	
	Hematoma/vacuolar hepatopathy (3)	Histopathology (1)	1 cat
Kidney (2)	Mesenchymal tumor (1)	Histopathology (1)	
	Bilateral polycystic kidney disease (1)	Histopathology (1)	
Caecum (1)	Hemangiosarcoma (1)	Histopathology (1)	

**Table 4:** Origin, diagnosis and outcomes of 21 cats with spontaneous hemoperitoneum undergoing conservative management.

Bleeding Origin	Diagnosis	Method of Diagnosis	Not Surviving to Discharge
Liver (14)	Amyloidosis (6)	Cytology (6)	3 cats
	Hepatocellular carcinoma (1)	Cytology (1)	
	Hepatopathy/neutrophilic inflammation (2)	Cytology (1), post-mortem examination (1)	1 cat
	Hemangiosarcoma (1)	Post-mortem examination (1)	
	Nodular hyperplasia/cholangiohepatitis (1)	Cytology (1)	1 cat
	Hepatic lipidosis (1)	Post-mortem examination (1)	1 cat
	Hematoma (1)	Cytology (1)	
	Unknown hepatic neoplasia (1)	Cytology (1)	
Spleen (5)	Hemangiosarcoma (4)	Cytology (3), post-mortem examination (1)	3 cats
	Mast cell tumor (1)	Cytology (1)	1 cat
Pancreas (2)	Pancreatic carcinoma (1)	Cytology (1)	1 cat
	Chronic pancreatitis (1)	Cytology (1)	

**Table 5:** Comparison of clinical parameters between survival outcomes and surgical status in cats with spontaneous hemoperitoneum.

	Surgery (22 Cats)	No Surgery (21 Cats)	*p*-Value
Age	9.5 years (6–12)	9 years (5–12)	0.714
Gender	10/22 females (45.5%) 12/22 males (54.5%)	10/21 females (48%) 11/21 males (52%)	0.610
Body weight	4.2 kg (4.1–5)	3.75 (3.2–5.5)	0.612
Body condition score	5 (4–5)	4 (3–5)	0.118
**Collapsing episodes**	**11/22 (50%)**	**3/21 (14%)**	**0.023**
Respiratory signs	2/22 (9%)	2/21 (9.5%)	0.659
Tachycardia	4/20 (20%)	4/18 (22%)	0.589
Duration clinical signs	2.5 days (1–3)	3.5 days (2–6)	0.087
Comorbidities	3/22 (14%)	6/21 (28%)	0.181
Shock index	2.00 (1.37–2.22)	1.84 (1.67–2.05)	0.796
Packed cell volume	13.5% (12–17)	16% (11–17)	0.630
Hypoalbuminemia	6/16 (37%)	4/16 (25%)	0.450
**Received transfusion**	**19/22 (86%)**	**10/21 (48%)**	**0.013**
Bleeding origin (spleen vs. liver)	10 vs. 9	5 vs. 14	0.092
Year of presentation (2005–2016 vs. 2017–2024)	10 vs. 12	11 vs. 10	0.441
**Survive to discharge**	**18/22 (82%)**	**10/21 (48%)**	**0.031**
	**Survived to discharge (28 cats)**	**No survived to discharge (15 cats)**	***p*** **-value**
Age	9.5 years (7.5–11.5)	9 years (4–12)	0.436
Gender	12/28 females (43%) 16/28 males (57%)	8/15 females (53%) 7/15 males (47%)	0.455
Body weight	4.6 kg (3.8–5.0)	4.4 kg (3.3–5.6)	0.310
Body condition score	5 (4–6)	4 (3–5)	0.144
Collapsing episodes	10/28 (36%)	2/15 (13%)	0.138
Respiratory signs	3/28 (11%)	1/15 (7%)	0.593
Tachycardia	3/25 (12%)	5/13 (38%)	0.094
Duration clinical signs	3 days (2–10)	2 days (1–7)	0.501
Comorbidities	6/28 (21%)	3/15 (14%)	0.645
Shock index	1.91 (1.41–2.22)	1.88 (1.52–2.20)	1.000
Packed cell volume	15% (11.5–19.5)	16% (14–18)	0.589
Hypoalbuminemia	5/21 (24%)	5/9 (55%)	0.104
Received transfusion	20/28 (71%)	9/15 (60%)	0.447
Bleeding origin spleen vs. liver	8 vs. 15	10 vs. 7	0.483
Year of presentation (2005–2016 vs. 2017–2024)	14 vs. 14	7 vs. 8	0.455
**Surgery**	**18/28 (64%)**	**4/15 (26%)**	**0.031**
Diagnosis * neoplasia vs. no neoplasia	18 vs. 9	6 vs. 5	0.366

Categorical variables are described as frequency and percentages whereas continuous variables are described as median and interquartile range (IQR). Variables highlighted in bold are considered significant at *p* < 0.05. * Diagnosis based on histopathology and cytology results.

## Data Availability

The data presented in this study are available from the corresponding author upon reasonable request. Restrictions apply to the availability of these data due to privacy considerations and compliance with the General Data Protection Regulation (GDPR).

## Abbreviations

The following abbreviations are used in this manuscript:

ALT	Alanine aminotransferase
ALP	Alkaline phosphatase
HSA	Hemangiosarcoma
IQR	Interquartile range
PCV	Packed cell volume
pRBC	Packed red blood cells
SD	Standard deviation
SI	Shock index
WBC	White blood cells
